# Racial differences in upper airway collapsibility and loop gain in young adult males

**DOI:** 10.1093/sleep/zsad091

**Published:** 2023-03-31

**Authors:** Shipra Puri, Gino S Panza, Dylan Kissane, Steven Jones, Kevin Reck, Ho-Sheng Lin, M Safwan Badr, Jason H Mateika

**Affiliations:** Research and Development, John D. Dingell Veterans Affairs Medical Center, Detroit, MI 48201, USA; Department of Physiology, Wayne State University School of Medicine, Detroit, MI 48201, USA; Research and Development, John D. Dingell Veterans Affairs Medical Center, Detroit, MI 48201, USA; Department of Health Care Sciences, College of Pharmacy and Health Science, Detroit, MI 48201, USA; Research and Development, John D. Dingell Veterans Affairs Medical Center, Detroit, MI 48201, USA; Department of Physiology, Wayne State University School of Medicine, Detroit, MI 48201, USA; Research and Development, John D. Dingell Veterans Affairs Medical Center, Detroit, MI 48201, USA; Department of Physiology, Wayne State University School of Medicine, Detroit, MI 48201, USA; Research and Development, John D. Dingell Veterans Affairs Medical Center, Detroit, MI 48201, USA; Department of Physiology, Wayne State University School of Medicine, Detroit, MI 48201, USA; Research and Development, John D. Dingell Veterans Affairs Medical Center, Detroit, MI 48201, USA; Department of Otolaryngology, Wayne State University School of Medicine, Detroit, MI 48201, USA; Research and Development, John D. Dingell Veterans Affairs Medical Center, Detroit, MI 48201, USA; Department of Physiology, Wayne State University School of Medicine, Detroit, MI 48201, USA; Department of Internal Medicine, Wayne State University School of Medicine, Detroit, MI 48201, USA; Research and Development, John D. Dingell Veterans Affairs Medical Center, Detroit, MI 48201, USA; Department of Physiology, Wayne State University School of Medicine, Detroit, MI 48201, USA; Department of Internal Medicine, Wayne State University School of Medicine, Detroit, MI 48201, USA

**Keywords:** loop gain, airway collapsibility, racial differences

## Abstract

**Study Objectives:**

Previous studies reported that the apnea–hypopnea index was similar in young adult Black and White participants. However, whether this similarity reflects an analogous combination of apneas and hypopneas is unknown. Likewise, the physiological mechanisms underlying this similarity has not been explored.

**Methods:**

60 Black and 48 White males completed the study. After matching for age and body mass index, 41 participants remained in each group. All participants completed a sleep study. Subsequently, standard sleep indices along with loop gain and the arousal threshold were determined. In addition, airway collapsibility (24 of 60 and 14 of 48 participants) and the hypoxic ventilatory response during wakefulness (30 of 60 and 25 of 48 participants) was measured.

**Results:**

The apnea–hypopnea index was similar in Blacks and Whites (*p* = .140). However, the index was comprised of more apneas (*p* = .014) and fewer hypopneas (*p* = .025) in Black males. These modifications were coupled to a reduced loop gain (*p* = .0002) and a more collapsible airway (*p* = .030). These differences were independent of whether or not the groups were matched. For a given hypoxic response, loop gain was reduced in Black compared to White males (*p* = .023).

**Conclusions:**

Despite a similar apnea–hypopnea index, more apneas and fewer hypopneas were evident in young adult Black compared to White males. The physiological mechanisms that contribute to these events were also different between groups. Addressing these differences may be important when considering novel therapeutic approaches to eliminate apnea in Black and White participants.

## Graphical Abstract

**Graphical Abstract:**
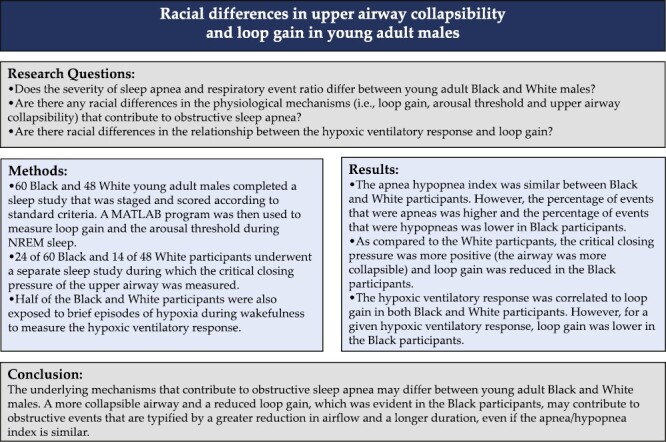

**Statement of Significance**

Demographic factors, such as age and race, are among the important variables that might impact the severity of obstructive sleep apnea and the coincident underlying mechanisms. In the present study, apnea severity and the concomitant underlying mechanisms were compared in Black and White young adult males. Our results showed that the severity of sleep apnea was similar in Black and White males. However, loop gain was reduced and the airway was more collapsible in young adult Black compared to White males. Addressing these differences may be important when considering novel therapeutic approaches to eliminate apnea in Black and White participants.

## Introduction

Obstructive sleep apnea is a disorder associated with persistent collapse or narrowing of the upper airway during sleep [1–3]. This disorder is associated with several detrimental outcomes including excessive daytime sleepiness, enhanced sympathetic nervous system activity, increased cardiovascular risk, and impaired cognition [1, 4, 5]. The severity of sleep apnea, as defined by the apnea–hypopnea index, may varying in relation to sex, age, and race.

In regard to race, limited published data exploring differences in sleep apnea severity in Black compared to White participants has been equivocal [6–9]. In some cases, no difference was reported [6], while other studies indicated that the severity of sleep apnea was greater in Black compared to White males [7]. This lack of certainty could be a consequence of the heterogeneity in the participants recruited for each study. On the other hand, the equivocal nature of the findings might be related to the influence of other variables. Indeed, Redline and colleagues [8] initially reported that differences in severity between Blacks and Whites manifest more clearly in younger individuals less than 25 years of age. Conversely, the severity of sleep apnea was similar between races up to approximately 50 years of age before a decline in severity was evident in elderly Black compared to White participants [8]. Redline and colleagues [8] suggested that differences in anatomy (i.e. soft tissue and nasopharyngeal volumes, and tonsillar hypertrophy) that predominantly influence airway patency at a younger age may have been responsible for the reported racial differences.

This speculation highlights the likelihood that underlying physiological mechanisms, that differ across race and manifest at different ages, could explain racial disparities in breathing instability. Published findings indicate that there are at least four principal endotypes that might contribute to sleep apnea [1, 10, 11]. Increased collapsibility of the upper airway, a blunted upper airway muscle response to changes in the partial pressure of carbon dioxide, a modified arousal threshold, and instability of the ventilatory control system (i.e. increased loop gain) [1, 10, 11]. The possibility that these endotypes differ across race, and that the combined impact of these endotypes may vary across the lifespan, is supported by the work of Borker and colleagues [12]. These investigators examined differences in apnea–hypopnea characteristics between a group of elderly (i.e. 69 years of age on average) Black and White participants. These authors reported that the hypopnea duration was reduced in Black compared to White participants [12]. This reduction was coupled to reductions in loop gain, circulatory delay, and an increased passive upper airway collapsibility in Black compared to White participants [12]. One or more of these differences in endotypic characteristics could be responsible for the variation in hypopnea duration.

Based on the published findings outlined above, the present investigation was designed to extend previous results and explore differences in the severity of sleep apnea, in a group of primarily young adult Black and White males. Based on Redline and colleagues’ [8] previous findings, we hypothesized that the apnea–hypopnea index in this age range would be similar. However, because it is becoming increasingly evident that the apnea–hypopnea index is not be the sole metric of obstructive sleep apnea severity [13], we proposed that other potential indices of breathing instability (e.g. the % of apneas and hypopneas that comprise the apnea–hypopnea index and event duration) would vary between Black and White males. Moreover, we proposed that racial differences would also be evident in the underlying mechanisms responsible for breathing instability, as previously reported (see Discussion section for details) [8, 12, 14, 15]. Lastly, as a corollary to these primary hypotheses, we determined if the hypoxic ventilatory response measured during wakefulness was correlated to loop gain measured during sleep, as implied by previous investigations (see Discussion section for details) [16–19]. We extended this exploration to determine if racial differences were evident in the correlation between the hypoxic ventilatory response measured during wakefulness and loop gain measured during sleep.

## Methods

### Participants

Data was obtained from Black (*n* = 60) and White (*n* = 48) male participants that were enrolled in studies completed in our laboratory between the years 2009 and 2022 [20–27]. This data will be shared upon reasonable request to the corresponding author. In each investigation, in-lab baseline sleep studies were completed, while participants were in the supine position, to confirm the presence of sleep apnea. Participants were required to have an apnea–hypopnea index >5 events/hour. In all cases, participants were not treated with medication and were not treated with continuous positive airway pressure prior to completing the baseline study. In addition, all participants had normal lung function (forced vital capacity >80% of predicted values; FEV_1.0_/FVC > 70% of predicted values), a normal sleep–wake cycle (i.e. no shift work) and a sleep efficiency greater than 75%. The majority of participants did not have any associated co-morbidities (*n* = 76) and the remaining participants (*n* = 32) were living with untreated hypertension (blood pressure >130/80 mm Hg) without other accompanying co-morbidities. A small (~6 years on average) but statistically significant difference in age existed between the Black and the White participants (see Results section). Thus, we matched Black (*n* = 41 of 60) and White (*n* = 41 of 48) participants based on age and body mass index to confirm that our findings were not age related. The age and body mass index range used for matching was ± 3 years and ± 3 kg/m^2^, respectively.

### Baseline sleep studies

During the baseline sleep study, electroencephalograms (EEG) (C3/A2, C4/A1, O1/A2, O2/A1), electrooculograms, submental electromyography, and a three-lead electrocardiogram were recorded. Chest wall and abdominal movements were recorded using inductive plethysmography (Respitrace, Ambulatory Monitor, Inc., Ardsley, NY, USA). Airflow and tidal volume were measured using a pneumotachometer (Model RSS100-HR, Hans Rudolph Inc., Kansas, MO, USA) attached to a tight-fitting face mask. Upper airway pressure was also measured using a transducer tipped catheter (MPC-500, Millar, Inc., Houston, TX, USA). The catheter was inserted nasally until the tip of the catheter was above the base of the tongue but below the uvula. Heart rate and oxygen saturation were recorded using a pulse oximeter (BIOX 3700, Ohmeda Corp., Laurel, MD, USA). All variables were converted from analog to digital at a sampling frequency of 100 Hz per channel and input into a commercially available software package (Gamma v. 4.0, Astro-Med Inc., West Warwick, RI, USA).

Standard criteria were used for scoring sleep stages and identifying breathing events [28]. Cortical arousals and breathing events were normalized to sleep time. Apneas were characterized by a greater than 90% reduction in airflow for a minimum of 10 s, while a hypopnea was characterized by a greater than 30% reduction in airflow for at least 10 s. The start of events was marked from the nadir of the last non-flow limited breath until the restoration of flow. If an event occurred immediately after a cortical arousal, thus making the preceding non-flow limited breath during wakefulness, then the start of the event was from the nadir of the first inspiration once sleep was established. All events were marked if there was an arousal. If an arousal was not present, the breathing event required a 3% or greater reduction in oxygen saturation. Cortical arousals were marked from the onset and offset of the arousal using the standard criteria of a significant change in EEG amplitude and/or frequency lasting at least 3 s. The end of the arousal was determined when a subsequent change in EEG amplitude or frequency was evident.

### OSA endotypes

A customized MATLAB (Mathworks, Natick, MA, USA) program, that has been previously described [29–31], was used to measure loop gain (see next paragraph for list of components) and the arousal threshold from the baseline sleep studies. Seven-minute windows of non-rapid eye movement (NREM) sleep were identified by the program. The dominant sleep stage for each window (i.e. the sleep stage that encompassed greater than 50% of the window) was determined. Seven-minute windows without a dominant sleep stage were excluded from data analysis. The 7-minute window duration was chosen to provide time for approximately 10 cyclic obstructive events to occur. The number of potential obstructive events was based on an average between event interval of approximately 40 s. This interval was considered sufficient to separate the chemical drive from the arousal contribution to ventilatory output during and following an obstructive event.

A breath-by-breath time series was generated for each 7-minute window and those breaths associated with an EEG arousal and/or flow limitation were identified. The customized program was employed subsequently to measure loop gain and the arousal threshold. Briefly, development of the program was based on the premise that the ventilatory control system is disturbed during breathing events and as a result carbon dioxide increases and oxygen decreases leading to an increase in ventilatory drive. The increase in drive is reflected by the level of hyperventilation that is evident when the airway opens after termination of an obstructive event. Consequently, the ventilatory drive was modeled as the sum of the ventilatory response to chemical stimuli (i.e. increases in carbon dioxide and decreases in oxygen that accompany apnea and hypopnea) and arousal [31]. The time course of the ventilatory response to chemical stimuli was modeled using parameters that reflect the circulation time between the lung and chemoreceptors (i.e. time delay), the time course of carbon dioxide buffering in the lung and tissues (i.e. time constant) and the overall sensitivity of the response (i.e. gain) [31]. These parameters along with the ventilatory response to arousal were modified until the modeled ventilatory drive closely fit the ventilation measured when the airway was not obstructed. These parameters were then used to calculate the magnitude of loop gain at the natural frequency (LG_n_) of obstructive events and at a selected frequency that is typical of the timing of obstructive events. To be consistent with the timing of apneic events and other published findings, loop gain was measured at a selected frequency of 1 cycle/minute (LG_1_) (i.e. 60 events/hour) for each 7-minute window [31]. For each window, the ventilatory drive immediately prior to the start of each scored EEG arousal (e.g. at the termination of a respiratory event) was identified. The mean value of these ventilatory drive values was deemed to be the arousal threshold.

The time delay, time constant, loop gain, arousal threshold, and the ventilatory response to arousal measured during NREM sleep were averaged for each participant. To evaluate the overall timing properties of the feedback response we also quantified the natural cycling period, which is defined as the duration of the periodic breathing cycle. A higher natural cycling period indicates a slower chemical response to ventilatory stimuli.

### Critical closing pressure

In addition to completing baseline sleep studies we also obtained measures of upper airway collapsibility (i.e. the critical closing pressure) [21, 25], in some Black (*n* = 24 of 60) and White (*n* = 14 of 48) participants during a separate sleep study. Airway patency was initially maintained using a holding pressure that was 1 cm H_2_O below the therapeutic pressure. The holding pressure was employed to prevent overdistension of the airway. Measurement of the critical closing pressure was performed by reducing the mask pressure in a stepwise fashion by increments of 1–2 cm H_2_O (Pcrit 3000, version 1.0; Philips Respironics, Murrysville, PA) for a duration of three to five breaths. Each step down in pressure was separated by a 1-min recovery period at the holding pressure. Stepwise reductions in pressure continued until the airway was fully collapsed, which was defined by measures of airflow <10% of baseline.

If stable sleep could not be maintained due to arousals in response to large decreases in pressure, airway collapsibility was determined by extrapolating the data from the relationship between pressure and flow obtained from those step downs in which a decrease in pressure was not accompanied by arousal [21, 25]. Specifically, inspiratory flow for each breath was obtained and averaged for each step down. Thereafter, the average flow was plotted against the pressure and a best fit line was used to extrapolate flow to zero. A linear and exponential curve were fit to the data. The curve with the best fit was used to determine the pressure in which an apnea would have occurred.

### Hypoxic ventilatory response

Black (30 of 60) and White (25 of 48) participants were also acutely exposed during wakefulness to 12 episodes of mild intermittent hypoxia that were 2–4 min in duration [23–26]. The episodes were interspersed with periods of normoxia of a similar length (see references [23–26] for description of the protocol). During each episode, the partial pressure of oxygen was maintained at 50 mm Hg, corresponding to an oxygen saturation of ~ 87–88%. Throughout the protocol the partial pressure of carbon dioxide was maintained slightly above baseline values (i.e. 2–3 mm Hg). The hypoxic ventilatory response (see below for calculation) was determined by averaging the response to an episode of hypoxia at the beginning and end of the protocol.

To quantify the hypoxic ventilatory response, the increase in minute ventilation from baseline to the second hypoxic episode and second to last hypoxic episode was divided by the change in the partial pressure of end-tidal oxygen from baseline to the second and second to last episode of the protocol. The ventilatory responses to the two hypoxic episodes were averaged.

### Statistical analysis

Statistical comparisons between Black and White participants were completed using an unpaired *t*-test. If the data was not normally distributed a simple transformation (i.e. square root) of the data was completed prior to completing the *t*-test. If the data was not normally distributed following transformation of the data, a Mann–Whitney Rank Sum Test was completed. A Pearson correlation coefficient analysis was used to examine the relationship between the hypoxic ventilatory response and loop gain. A *t*-test was used to compare the slope of the regression lines fit to the data obtained from the Black and White participants. A Pearson correlation coefficient analysis was also used to determine the correlation between loop gain and the average frequency of events measured from the 7-minute windows.

## Results

The Black (*n* = 60) participants were slightly older than the White (*n* = 48) participants (Table 1). In contrast, the body mass index was similar between groups (Table 1). No difference in age and body mass index was evident after matching the participants on the basis of these variables (Table 2). No difference in sleep architecture was evident between groups, with the exception that the duration of time spent in N3 was less in Black compared to White participants (Tables 1 and 2).

**Table 1.** Demographics and sleep architecture of the study participants

Variable	Black (*n* = 60)			White (*n* = 48)			*P* value
	Mean	Median	IQR	Mean	Median	IQR	
Age (years)	34.9	33.0	26.0–40.8	28.7	27.5	23.0–33.0	.002
BMI (kg/m^2^)	27.8	27.0	24.9–30.3	28.1	28.0	26.0–30.0	.360
TST (min)	328.8	334.0	301.4–369.3	315.7	312.5	280.4–345.3	.256
SE (%)	78.6	79.3	73.6–86.9	76.6	78.5	71.5–82.7	.302
N1 (%)	53.4	50.1	33.8–76.4	46.0	50.5	24.8–63.0	.139
N2 (%)	39.4	41.3	21.3–56.0	45.1	44.6	25.1–66.3	.253
N3 (%)	2.3	0.0	0.0–2.6	4.6	5.8	0.0–7.8	.001
REM (%)	5.0	2.9	0.0–7.1	4.3	2.2	0.0–6.1	.990

BMI, body mass index; TST, total sleep time; SE, sleep efficiency; REM, rapid eye movement; IQR, interquartile range.

**Table 2.** Demographics and sleep architecture of the study participants (age and BMI matched)

Variable	Black (*n* = 41)			White (*n* = 41)			*P* value
	Mean	Median	IQR	Mean	Median	IQR	
Age (years)	29.9	28.0	24.0–33.5	28.0	25.0	22.5–31.0	.243
BMI (kg/m^2^)	27.1	27.0	24.9–28.1	27.4	27.0	26.0–29.4	.639
TST (min)	325.4	335.0	309.0–370.8	313.5	308.5	281.8–344.0	.386
SE (%)	78.5	80.2	72.9–89.3	76.2	78.5	71.2–82.7	.254
N1 (%)	49.0	45.8	29.5–64.5	44.4	44.5	23.6–59.0	.396
N2 (%)	42.8	46.1	31.4–59.3	46.3	45.1	26.6–67.3	.600
N3 (%)	2.7	0.0	0.0–4.6	5.0	5.8	0.2–8.0	.005
REM (%)	5.6	3.8	0.0–7.0	4.3	2.1	0.0–6.1	.814

BMI, body mass index; TST, total sleep time; SE, sleep efficiency; REM, rapid eye movement; IQR, interquartile range.

**Figure 1.**
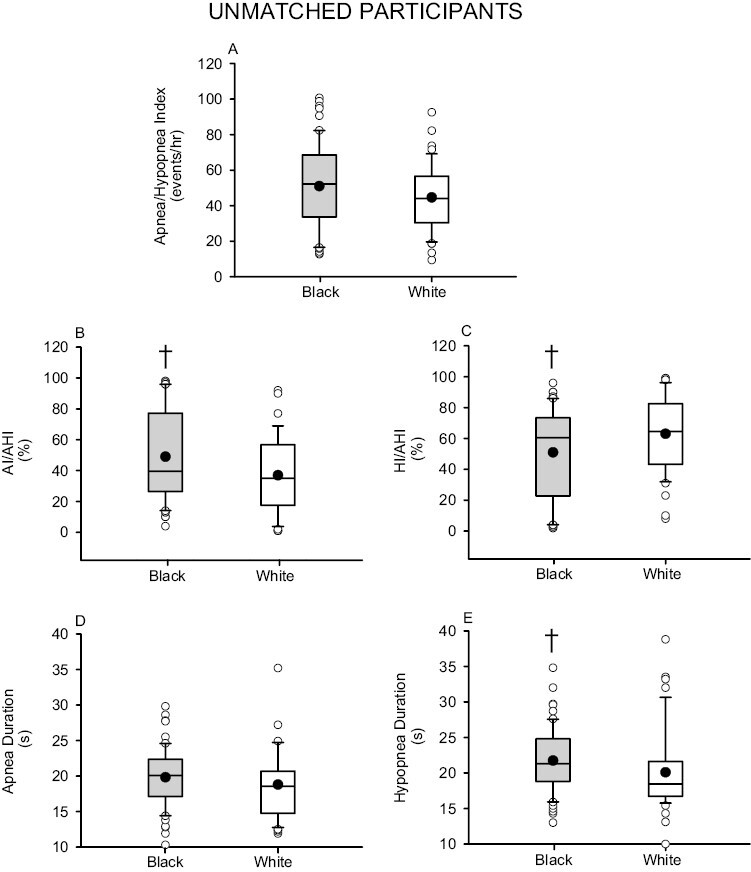
Box and whisker plots showing the mean (black dot) and median (black line) (A) apnea–hypopnea index (B) apnea index/apnea–hypopnea index ratio (AI/AHI) (C) hypopnea index/apnea–hypopnea index ratio (HI/AHI) (D) apnea duration and (E) hypopnea duration. Note that the upper and lower limits of the box indicate the 25% and 75% quartiles, respectively. In addition, the whiskers at either end of the box represent the greatest and least value excluding outliers. The white circles represent outliers that are 1.5 times the upper or lower quartile. These traits are the same for the remaining box and whisker plots shown in subsequent figures. Note that the AI/AHI ratio was higher and the HI/AHI ratio was lower in Black participants (gray boxes) (*n* = 60) compared to White participants (white boxes) (*n* = 48). Statistical differences between Black and White participants were determined using an unpaired *t*-test or a Wilcoxon rank sum test. ^†^Significantly different from White participants.

**Figure 2.**
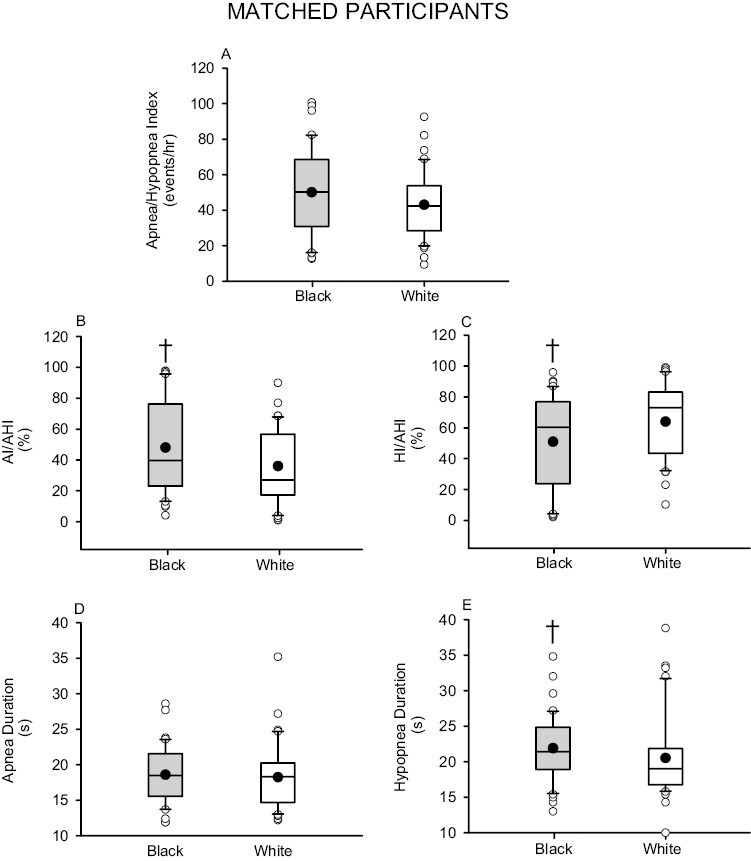
Box and whisker plots showing the mean (black dot) and median (black line) (A) apnea–hypopnea index (B) apnea index/apnea–hypopnea index ratio (AI/AHI) (C) hypopnea index/apnea–hypopnea index ratio (HI/AHI) (D) apnea duration and (E) hypopnea duration. Note that the AI/AHI ratio was higher and the HI/AHI ratio was lower in Black participants (gray boxes) (*n* = 41) compared to White participants (white boxes) (*n* = 41) matched for age and body mass index. Statistical differences between Black and White participants were determined using an unpaired *t*-test or a Wilcoxon rank sum test. ^†^Significantly different from White participants.

The apnea–hypopnea index was similar in Black and White participants (before matching, *p* = .127; after matching, *p* = .140) (Figures 1A and 2A). Despite this similarity, the apnea/apnea–hypopnea ratio was greater in Black compared to White participants (before matching, *p* = .044; after matching, *p* = .014) (Figures 1B and 2B). Conversely, the hypopnea/apnea–hypopnea ratio was lower in Black compared to White participants (before matching, *p* = .043; after matching, *p* = .025) (Figures 1C and 2C). The apnea duration was similar in Black and White participants (before matching, *p* = .201; after matching, *p* = .660) (Figures 1D and 2D). However, the hypopnea duration was greater in Black compared to White participants (before matching, *p* = .010; after matching, *p* = .039) (Figures 1E and 2E).

**Figure 3.**
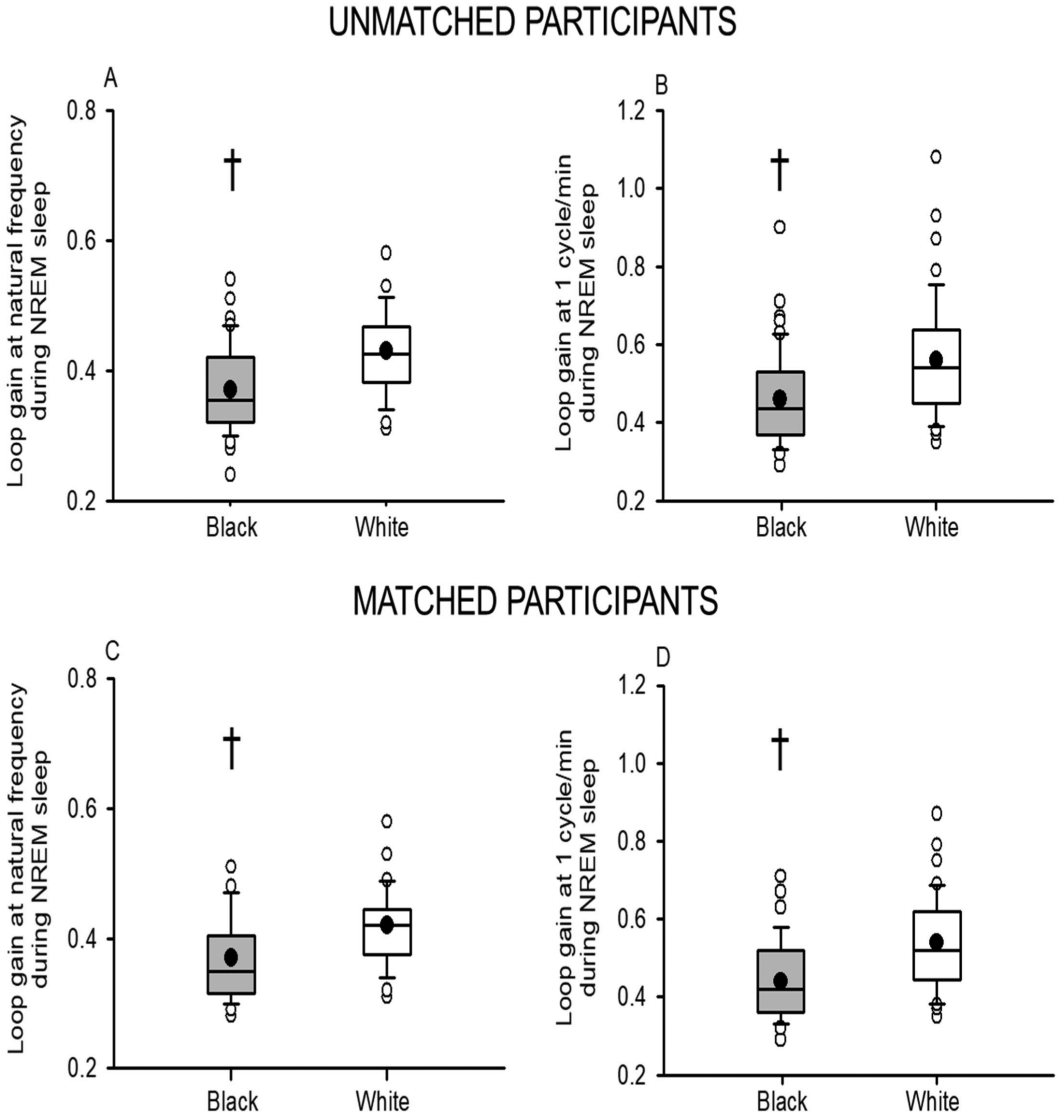
Box and whisker plots showing the mean (black dot) and median (black line) natural loop gain before (A) and after (C) matching. Similar plots showing loop gain at 1 cycle per minute before (B) and after (D) matching. Note that loop gain was lower in Black participants (gray boxes) before (*n* = 60) and after matching (*n* = 41) compared to White participants (white boxes) before (*n* = 48) and after matching (*n* = 41). Statistical differences between Black and White participants were determined using an unpaired *t*-test or a Wilcoxon rank sum test. ^†^Significantly different from White participants.

The disparity in the apnea/apnea–hypopnea and hypopnea/apnea–hypopnea ratios evident in Black compared to White participants was accompanied by a decrease in loop gain in Black participants (before matching, LGn, *p* = .00001 and LG1, *p* = .001; after matching, LGn, *p* = .0001 and LG1, *p* = .0002) (Figure 3A–D). In addition, loop gain was not correlated to the average number of events recorded during each 7-minute window in the Black participants (LG_1_: *R* = 0.09, *p* = .489) but a significant correlation was evident in the White participants (LG_1_: *R* = 0.47, *p* = .0007).

**Figure 4.**
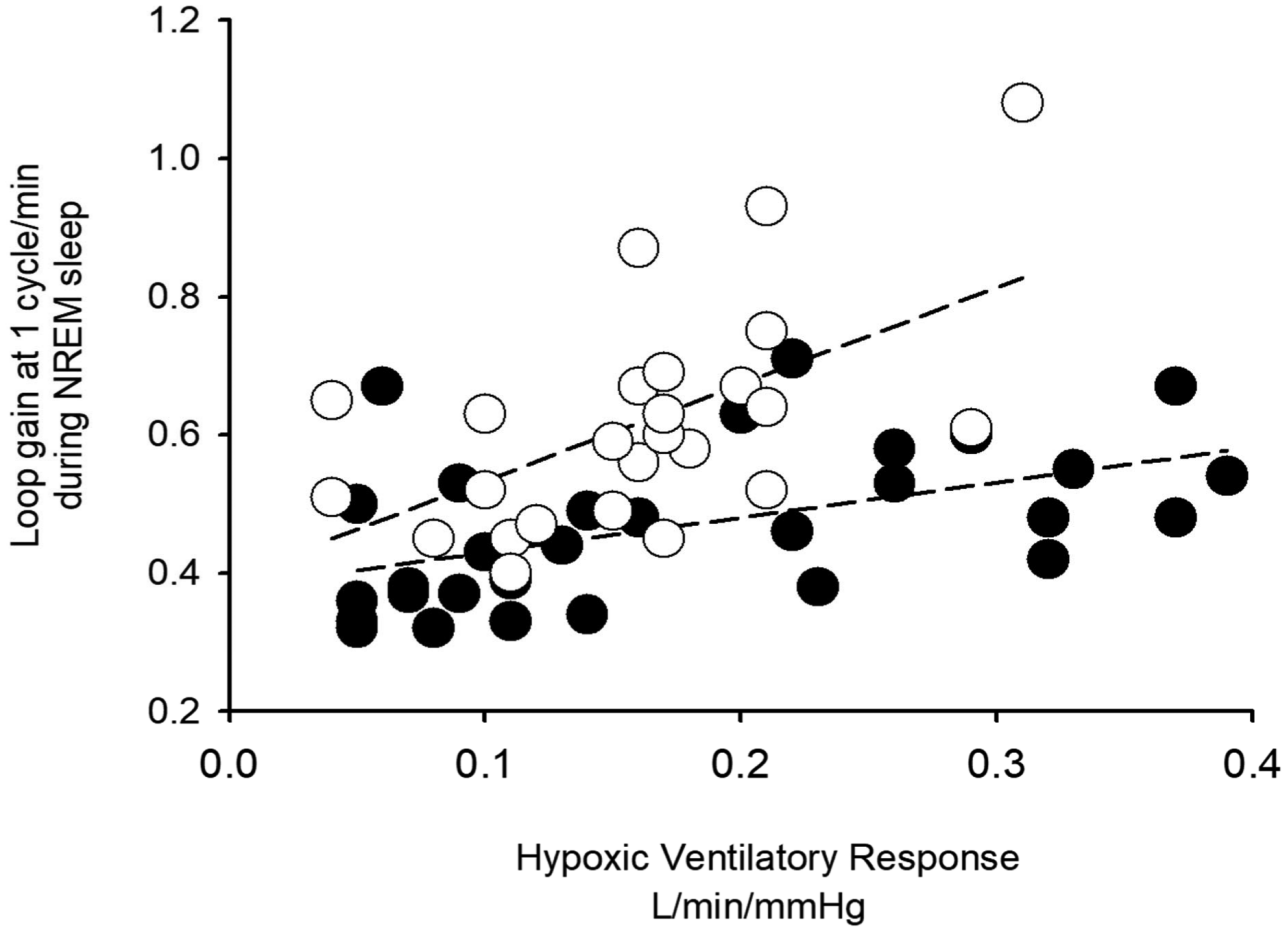
Scatterplot showing the relationship between the hypoxic ventilatory response measured during wakefulness and loop gain measured during non-rapid eye movement sleep in Black (black circles) (*n* = 30) and White (white circles) (*n* = 25) participants. Note that the slope of the regression line was reduced in Black compared to White participants. Consequently, for a given hypoxic ventilatory response, loop gain was reduced in Black compared to White participants. Pearson correlation analysis was used to determine if the hypoxic ventilatory response and loop gain were correlated. A linear regression analysis was used to model the relationship and a *t*-test was used to compare the slopes of the regression line.

The hypoxic ventilatory response during wakefulness was correlated to loop gain in both the Black and White participants (LG_1_—Black *R* = 0.50, *p* = .005; White *R* = 0.57, *p* = .003) (Figure 4). The slope of the regression line was reduced in Black compared to White participants (*p* = .023). Thus, for a similar hypoxic ventilatory response, loop gain during NREM sleep was reduced in Black compared to White participants (Figure 4).

**Figure 5.**
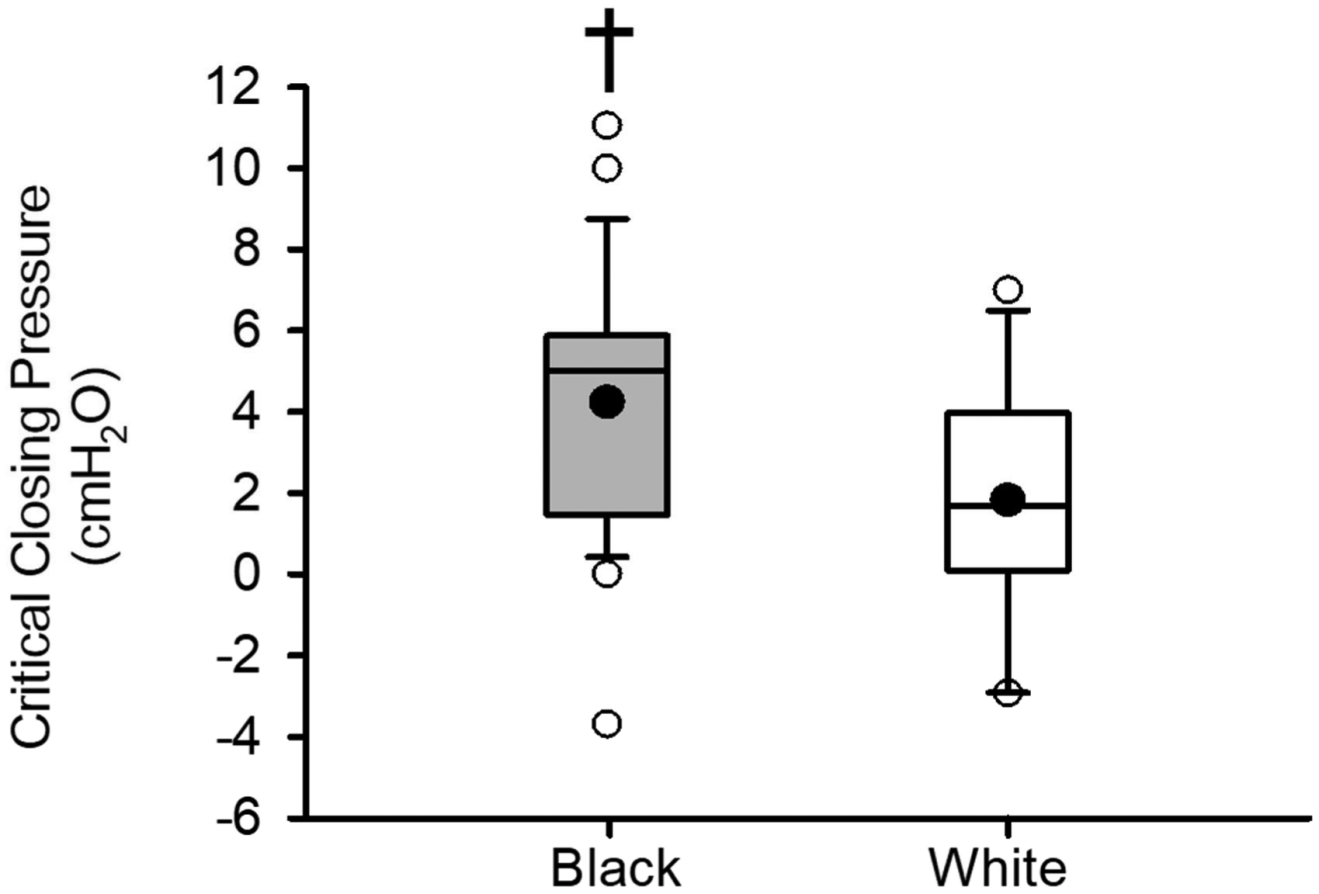
Box and whisker plots showing the mean (black dot) and median (black line) critical closing pressure. Black participants (gray boxes) (*n* = 24) and White participants (white boxes) (*n* = 14). Statistical differences between Black and White participants were determined using an unpaired *t*-test or a Wilcoxon rank sum test. ^†^Significantly different from White participants.

**Figure 6.**
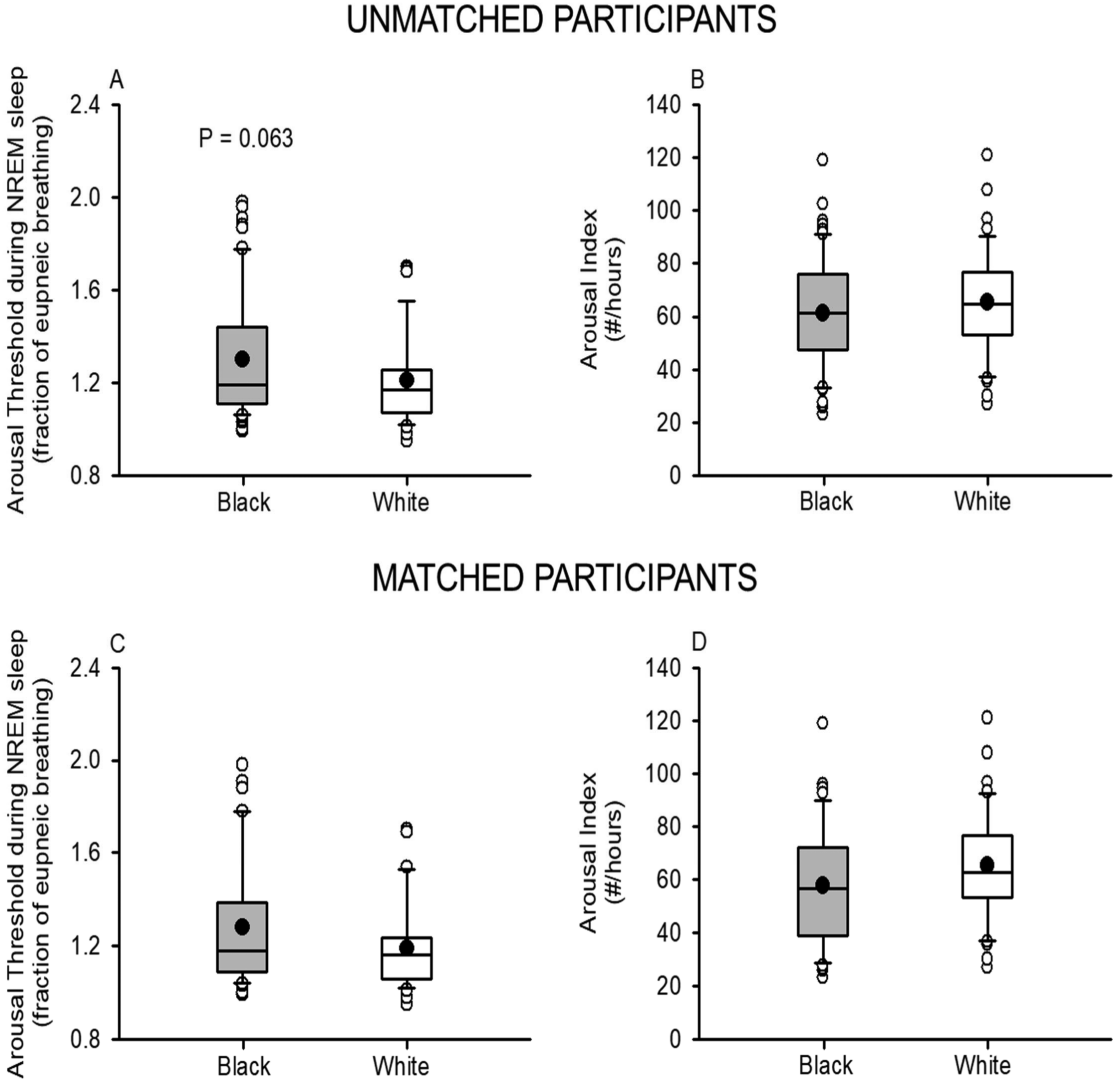
Box and whisker plots showing the mean (black dot) and median (black line) arousal threshold before (A) and after (C) matching. In addition, similar plots of the arousal index before (B) and after (D) matching are shown. Note that the arousal threshold and index was similar in Black participants (gray boxes) before (*n* = 60) and after matching (*n* = 41) compared to White participants (white boxes) before (*n* = 48) and after matching (*n* = 41). Statistical differences between Black and White participants were determined using an unpaired *t*-test or a Wilcoxon rank sum test. ^†^Significantly different from White participants.

The decrease in loop gain was also associated with a more positive critical closing pressure (i.e. a more collapsible airway) in Black compared to White participants (*p* = .030) (Figure 5). In addition, a tendency toward an increase in the arousal threshold was evident in Black participants prior to matching (Figure 6A), although this difference did not reach statistical significance (*p* = .063). Following matching (Figure 6C), no clear difference in the arousal threshold (*p* = .117) was evident between groups. Lastly, the arousal index was similar in the Black and White participants (before matching, *p* = .302; after matching, *p* = .106) (Figure 6B and D).

## Discussion

Our results showed that the apnea–hypopnea index in young adult Black and White males were similar. However, more apneas and fewer hypopneas were evident in Black compared to White participants, when standardized to the apnea–hypopnea index. Our results also revealed that the duration of hypopneas was longer in Black compared to White participants.

Despite a similar apnea–hypopnea index, our results indicated that Black participants had a more collapsible airway and a lower loop gain compared to White participants. Our results also revealed that the hypoxic ventilatory response measured during wakefulness correlated to loop gain measures during sleep in both races. However, loop gain was reduced in Black compared to White participants, for a given hypoxic ventilatory response. Lastly, loop gain was not correlated to the number of events detected in Black participants but this correlation was evident in White participants.

### Methodological considerations

Race is considered to be a social construct. Thus, our findings showing differences between Black and White participants may not represent a specific biological variation. Rather the differences could be due to other unknown factors that existed between the groups studied in our investigation. We attempted to minimize these factors by ensuring that all participants were not treated with continuous positive airway pressure or other medications. Likewise, the majority of participants were healthy with the exception of living with obstructive sleep apnea. In those participants with hypertension, blood pressure was similar between groups and a similar number of participants with hypertension were included in the matched groups. Lastly, the age and body mass index were similar between groups. On the other hand, the effect of environment, diet, frequency of physical activity and years living with obstructive sleep apnea are a few unknown factors that could contribute to the noted differences. Thus, additional investigation is required.

It might be argued that examining differences in race could limit the generalizability of the findings to other populations. However, we do highlight, based on a comparison of data across studies [15] (see Mechanisms responsible for racial differences in obstructive sleep apnea section for details), that independent of race and age, groups characterized by a less collapsible airway, and increased loop gain appear to be accompanied by less severe forms of sleep apnea.

### Race and obstructive sleep apnea

Published data exploring differences in sleep apnea severity in Black compared to White participants has been equivocal [7–9, 32]. The equivocal nature of the findings could be related to a number of variables including age. Redline and colleagues [8] reported that differences in severity between Blacks and Whites manifest more clearly in younger individuals less than 25 years of age. In contrast, the severity of sleep apnea was similar between races up to approximately 50 years of age, before a decline in severity was evident in elderly Black compared to White participants [8]. The decline in severity in elderly Black particpants has been replicated in more recent studies [15]. In contrast, Ancoli and colleagues indicated that no racial differences were evident in older individuals (>55 years of age), while Pranathiageswaran and colleagues [7] reported that the apnea–hypopnea index was higher in Black compared to White males less than 39 years of age and between 50 and 59 years of age. Our results support the findings of Redline and colleagues, since no difference in the apnea–hypopnea index was found between young adult Black and White males in the present study.

Despite a similar apnea–hypopnea index, the novel result from our study showed that Black participants experienced more apneas and fewer hypopneas compared to White particpants, when expressed as a percentage of the apnea–hypopnea index. A significant increase in hypopnea duration was also evident in Black participants. It is unlikely that the difference in the percentage of apneic or hypopneic events was influenced by age, since this result remained after the groups were matched for this variable. Likewise, body mass index was similar between groups before and after participants were matched. Moreover, other potential factors that could be responsible for differences between the groups were minimized, given that the majority of sleep apnea participants were otherwise healthy and none of the participants were treated with medication. The increase in the apnea/apnea–hypopnea ratio indicates that young adult Black males might experience greater airway collapse and longer event duration compared to young adult White males, despite similarities in the apnea–hypopnea index.

### Mechanisms responsible for racial differences in obstructive sleep apnea

Limited published findings suggest that different mechanisms might contribute to obstructive events across racial groups. Previous work has shown that there are differences in cephalometric [8] and anthropometric [14] facial features in Black compared to White participants. The evidence indicates that the shape and size of skeletal components of the upper airway might have a more important role in airway collapsibility in Whites, while soft tissue structures might have greater importance in Black participants [14]. These differences could lead to variances in upper airway mechanics and collapsibility in Black compared to White participants. This contention is supported by the racial difference in respiratory event ratios (see Race and obstructive sleep apnea section), and by more direct evidence from our study, which revealed that the critical closing pressure was more positive (i.e. indicating a more collapsible airway) in Black compared to White participants.

It might be suggested that factors other than race were responsible for the difference in the critical closing pressure, since comparisons were only made between the unmatched groups. A comparison between matched groups was not completed because a limited sample size (*n* = 4 in each group) was available following matching. However, differences in the percentage of apneic events standardized to the total apnea–hypopnea index remained after matching, indicating that airway collapsibility might have played a predominate role in the increased percentage of apneic events that we observed in the Black participants. Exposure to more severe forms of airway collapse might result in increased morbidity and mortality despite similarities in the apnea–hypopnea index and the prevalence of sleep apnea.

In contrast to our findings, two recent studies reported that Black compared to White participants had a less collapsible airway. Measures of collapsibility were obtained using a less invasive technique then the one employed in the present study [12, 15]. One possible explanation for the discrepant findings is that airway collapsibility measures were obtained from an elderly population (average age 69 years old) compared to our sample (average age 32 years old). This reduced collapsibility could contribute to a reduction in apnea severity and breathing event duration that has been reported in elderly Black compared to White participants [12, 15].

In addition to a less collapsible airway, variations in loop gain in elderly Black compared to White participants have been reported [12, 15]. Initially, Borker and colleagues [12] reported that a reduction in loop gain, coupled to breathing events of shorter duration, was evident in elderly Black participants. Moreover, after adjusting for endotypic mechanisms racial differences in event duration were eliminated [12]. In a subsequent investigation, that employed a similar population (i.e. elderly Black and White participants), a reduction in airway collapsibility was coupled to an increase in loop gain in elderly Black participants, rather than a reduction [15]. The reduced collapsibility and increased loop gain were associated with a reduction in the apnea–hypopnea index in elderly Black versus White participants [15]. The results obtained from our younger cohort are in direct contrast to these latter findings, since collapsibility of the airway was increased and loop gain was reduced in Black compared to White participants. As mentioned, these discrepancies are likely linked to the younger age of our Black participants compared to the elderly Black participants studied previously. Despite the endotypic mechanistic differences in young versus elderly Black and White participants, it is interesting to note that independent of race and age, the groups characterized by a less collapsible airway and increased loop gain (i.e. elderly Black participants in Sands and colleagues’ study [15] and young White participants in the present study) were accompanied by less severe forms of sleep apnea, in regard to frequency and duration of events.

Based on the difference in loop gain that was evident in the Black and White participants, we determined if this variable was correlated to the average number of events measured throughout the 7-minute windows used to determine loop gain. Interestingly, our results showed that loop gain was not correlated to the number of events in the Black participant group. In contrast, a significant correlation was evident between loop gain and number of events in the White participants. Although the correlation analysis does not establish cause and effect, one possible interpretation of our results is that loop gain had a more predominant role in determining the number and duration of breathing events in White compared to Black participants.

Based on findings from previous studies [16–19], we were also interested in determining if loop gain measures obtained during sleep were correlated to measures of the hypoxic ventilatory response during wakefulness. Likewise, we were interested in determining if racial differences in this correlation were evident. Previous studies have reported that breath hold duration in healthy individuals is negatively correlated to measures of hypercapnic sensitivity during wakefulness [16–19]. Thus, chemoreflex sensitivity may have a predominant role in determining breath hold duration, although a number of other factors (i.e. initial lung volume, mechanical breathing movements) may also influence breath hold duration [16]. In the same vein, more recent findings reported that both controller gain (i.e. chemoreflex sensitivity) and loop gain (which is influenced in part by controller gain) during sleep was correlated to measures of breath hold duration during wakefulness in healthy individuals and individuals living with sleep apnea [16]. These findings were extended further to show that loop gain measured during both wakefulness and sleep were correlated in these participants [16]. Thus, it was not surprising that the hypoxic ventilatory response (i.e. a measure of chemoreflex sensitivity) measured during wakefulness was correlated to loop gain measures during sleep in our investigation. Besides confirming earlier findings, we also showed that the slope of the regression line fit to the hypoxic ventilatory response—loop gain relationship was significantly reduced in Black compared to White participants. In other words, for a given hypoxic ventilatory response during wakefulness, the loop gain measure during sleep was reduced in Black compare to White participants. Given the nature of the correlative relationship, further studies are required to address the mechanisms responsible for this racial difference. One possibility might be that increases in upper airway resistance and airway collapsibility are responsible for the reduced slope observed in the Black participants.

Lastly, our results did not reveal any significant differences in the arousal threshold in Black compared to White participants, when the groups were matched. This was somewhat surprising in that an increase in the arousal threshold was anticipated in Black compared to the White participants because of the increased percentage of events that were apneas and the increase in the hypopnea duration. Indeed, we have previously shown that the arousal threshold is positively correlated with the apnea–hypopnea index and apnea duration as well as negatively correlated with the percentage of events that are hypopneas [33]. Although differences were not evident between the matched groups, a difference in the arousal threshold approached statistical significance when the unmatched groups were compared. Our findings are similar to the results of Zinchuk et al [34] who reported that the arousal threshold was higher in Black compared to White participants. However, this difference was not statistically significant once a multivariate regression analysis, which included age, was completed. Thus, further investigation is required to determine if differences in the arousal threshold exist in adult Black and White participants independent of age.

## Conclusion

Our results showed that no difference in the apnea–hypopnea index was evident in a group of young adult Black and White males. However, we showed that a greater percentage of events were apneas and the duration of hypopneas was longer in Black compared to White participants. Our results showed that a lower loop gain and a more collapsible airway may have been responsible for the observed outcomes in the Black participants. Moreover, our findings revealed that the contribution of chemoreflex sensitivity (i.e. hypoxic ventilatory response measured during wakefulness) to loop gain measures might be less in Black compared to White participants. Addressing these differences may be important when considering novel therapeutic approaches to eliminate apnea in Black and White participants.
